# High Seroprevalence of Autoantibodies Typical of Autoimmune Liver Disease in Eastern Ethiopia: Is Chewing of Khat (Catha edulis) a Triggering Factor?

**DOI:** 10.1155/2018/4980597

**Published:** 2018-12-23

**Authors:** Stian Magnus Staurung Orlien, Tekabe Abdosh Ahmed, Nejib Yusuf Ismael, Nega Berhe, Trine Lauritzen, Svein Gunnar Gundersen, Asgeir Johannessen

**Affiliations:** ^1^Regional Advisory Unit for Imported and Tropical Diseases, Oslo University Hospital Ullevål, Oslo, Norway; ^2^Department of Paediatrics, Vestfold Hospital Trust, Tønsberg, Norway; ^3^Department of Internal Medicine, Jugal Hospital, Harar, Ethiopia; ^4^Haramaya University College of Health and Medical Sciences, Harar, Ethiopia; ^5^Department of Internal Medicine, Hiwot Fana Specialized University Hospital, Harar, Ethiopia; ^6^Aklilu Lemma Institute of Pathobiology, Addis Ababa University, Addis Ababa, Ethiopia; ^7^Department of Medical Biochemistry, Vestre Viken Hospital Trust, Drammen, Norway; ^8^Department of Global Development and Planning, University of Agder, Kristiansand, Norway; ^9^Department of Infectious Diseases, Vestfold Hospital Trust, Tønsberg, Norway

## Abstract

**Background:**

Recent studies have identified chewing of khat (*Catha edulis*) as an independent risk factor for liver injury; however, the pathogenetic mechanism remains poorly understood. Case series have found markers of autoimmune hepatitis in patients with khat-related liver disease, suggesting that khat chewing might trigger an autoimmune response. The aims of the present study were (i) to assess the prevalence of autoantibodies typical for autoimmune liver diseases in a healthy population in Ethiopia and (ii) to explore the hypothesis that khat usage triggers autoimmunity.

**Methods:**

Consenting adults (≥18 years) without known autoimmune disease or manifest liver disease were included. One-hundred-and-sixty-nine individuals with current khat use were compared to 104 individuals who never used khat. Seroprevalence of antinuclear (ANA), antismooth muscle (SMA), and antimitochondrial antibodies (AMA) were determined and compared between the groups using logistic regression models to adjust for age and sex.

**Results:**

Overall, 2.6% of the study subjects were positive for ANA, 15.4% for SMA, and 25.6% for AMA. When comparing khat users to nonusers, ANA was detected in 4.1% vs. 0% (p=0.047), SMA in 16.0% vs. 14.4% (p=0.730), and AMA in 24.9% vs. 26.9% (p=0.704). ANA was excluded from multivariable analysis since there was no seropositive in the reference group. After adjusting for sex and age, no significant association between khat use and SMA or AMA was found.

**Conclusions:**

No association between khat usage and the seropresence of SMA or AMA was found, weakening the hypothesis that khat-related liver injury is mediated through autoimmune mechanisms. However, the seroprevalences of AMA and SMA were strikingly high in this Ethiopian population compared to global estimates, suggesting that diagnostic algorithms for autoimmune liver diseases developed in Europe and North America might lead to misdiagnosis of patients on the African continent.

## 1. Introduction

*Catha edulis *(khat) is an evergreen shrub indigenous to East Africa and the Arabian Peninsula that is cultivated for its use as a natural stimulant [1]. Khat leaves contain more than 40 different compounds, including three alkaloids, cathinone (aminopropriophenone), cathine (norpseudoephedrine), and norephedrine, which are structurally related to amphetamine and exert a psychostimulatory effect on the central nervous system [2]. Fresh young leaves and twigs are chewed to increase performance, evoke alertness, and attain a state of euphoria enhancing social interaction [3]. The habit of khat chewing is common in East Africa and the Arabian Peninsula where it is considered as a part of the social and cultural heritage which has prevailed for ages, probably as far back as the 13^th^ century [4]. Over the last three decades, the Horn of Africa and Middle East have been engulfed by war and instability, leading to mass migration and hence a wider spread of khat consumption within immigrant communities worldwide [5]. In addition, there is a growing interest in plant-derived and uncontrolled psychoactive substances among youth in Europe and the USA [6, 7]. The global prevalence is unknown but estimates range up to 20 million daily users, which most likely is an underestimation [1].

Cathinone and cathine are controlled substances under the international Convention of Psychotropic Substances (1971), whereas fresh khat leaves are not. The World Health Organization has defined khat as a drug of abuse since it is associated with negative social and health consequences [8]. In addition to psychological adverse effects such as psychosis [9] and exacerbation of pre-existing psychotic disorder [10], khat use is associated with a wide range of somatic health problems, including acute and chronic liver disease [11].

In an earlier case-control study, we demonstrated a strong and significant association between khat chewing and chronic liver disease in Ethiopia [12]; however, the mechanism of liver injury was not addressed. A few previous case reports have described khat-related liver injury mimicking autoimmune hepatitis, and the authors speculate that khat might trigger an autoimmune reaction in susceptible individuals [13–15]. The hallmark of autoimmune hepatitis is the presence of certain autoantibodies, of which antinuclear antibodies (ANA) and antismooth muscle antibodies (SMA) are the most important. Other diagnostic criteria include elevated levels of immunoglobulin G (IgG), typical histopathological changes in the liver, and the absence of active viral hepatitis [16].

The prevalence of autoimmune disease and autoantibodies in Ethiopia is largely unknown. In an ancient study, Tsega et al. studied records from 7966 medical inpatients in four different hospitals in Addis Ababa between 1971 and 1978, of whom 0.2% had rheumatoid arthritis and 0.05% had systemic lupus erythematosus [17]. In a substudy of 107 Ethiopians with dyspepsia and 80 healthy controls recruited from 1975 to 1978, ANA was found in one (0.5%), SMA in 20 (10.7%), and antimitochondrial antibodies (AMA) in one (0.5%).

In the present study, which was nested in a previously published case-control study [12], we aimed to assess (i) the seroprevalence of autoantibodies typical of autoimmune liver diseases in a well-defined study population in Ethiopia and (ii) to explore the hypothesis that khat usage triggers autoimmunity. Results from this study might pave the road for a better understanding and ultimately better treatment of khat-related liver injury.

## 2. Material and Methods

### 2.1. Study Setting and Participants

A case-control study was undertaken at Hiwot Fana Specialized University Hospital and Jugal Hospital in Harar, Ethiopia, between April 2015 and April 2016, as previously described [12]. Study subjects for the present analysis were the controls from the previous study, and comprised of adults aged 18 years and above attending the ophthalmology, dermatology, or surgical services during the study period. Individuals with conditions associated with autoimmune markers were excluded from the analysis, such as (i) known human immunodeficiency virus infection, rheumatic or autoimmune disease; (ii) history of alcohol misuse, defined as >20 g/day in women and >30 g/day in men [18]; (iii) clinical signs or previous history of liver disease; or (iv) positive serum hepatitis B surface antigen (HBsAg) or hepatitis C virus antibody (anti-HCV).

### 2.2. Patient Assessment

All study participants underwent a semistructured interview by local nurses fluent in their mother tongue. Demographic data including age, sex, ethnicity, religion and occupation were recorded. Current diagnosis, previous medical history, alcohol drinking habits and use of herbal remedies and khat (*Catha edulis*) were explored.

**Figure 1:**
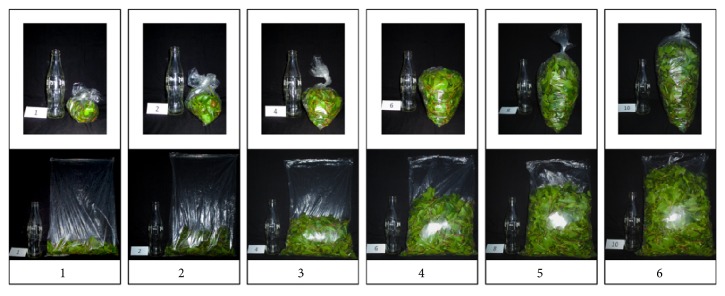
**The visual analogue scale used to quantify the use of khat in grams. (1)** 100 grams;** (2)** 200 grams;** (3)** 400 grams;** (4)** 600 grams;** (5)** 800 grams;** (6)** 1000 grams.

In lack of validated criteria for the quantification of khat usage, we established a screening tool to assess khat consumption as described in previous publications [12, 19]. By combining information on khat usage quantified in grams using a visual analogue scale (Figure 1) with the frequency and duration of khat usage categorized using the Drug Use Disorders Identification Test [20], we classified lifetime khat exposure as* khat-years*. Approximately 100-300 g of fresh khat leaves is chewed in a typical session [21]; thus one* khat-year* was defined as daily use of 200 g of fresh khat for one year.

Clinical examination was undertaken using a prespecified protocol. Study subjects with features suggestive of manifest liver disease, such as jaundice, ascites, hepatosplenomegaly, caput medusa, or spider angioma, were excluded from the analysis.

### 2.3. Laboratory Tests

Blood was collected by venous puncture for immediate processing; serum was separated for storage in aliquots at -20°C. Serum alanine aminotransferase (ALT) and aspartate aminotransferase (AST) activities were measured locally using a semiautomatic biochemistry analyser DR-7000D (DIRUI, Changchun, China) and HumaLyzer 3000 (HUMAN, Wiesbaden, Germany).

Validated rapid diagnostic tests were used to screen for HBsAg and anti-HCV; results were confirmed using enzyme-linked immunosorbent assays (ELISA) as previously described [12].

Serum specimens were transported on dry ice to Drammen Hospital in Norway and stored at -80°C until analysed. Autoimmune markers were determined by the Phadia™250 Laboratory system (Thermo Fisher Scientific, Waltham, MA, USA). ANA was detected using the EliA™ Symphony assay (Phadia, Freiburg, Germany) with a calculated ratio of test sample response to calibrator >1.0 defined as positive, 0.7-1.0 was equivocal, and <0.7 was negative [22, 23]; SMA was determined by QUANTA Lite® Actin IgG (Inova Diagnostics, San Diego, CA, USA) and a cut-off level >30 assay units was classified as positive, as proposed by the manufacturer [24]; AMA was determined using QUANTA Lite® M2 EP (MIT3) (Inova Diagnostics) and a cut-off level >25 assay units was classified as positive, as proposed by the manufacturer [25]. Serum was analysed for IgG using the IMMAGE® 800 Immunochemistry System (Beckman Coulter, Brea, CA, USA) and serum alkaline phosphatase (ALP) activity was measured using ARCHITECT ci16200 (Abbott Diagnostics, Abbott Park, IL, USA).

### 2.4. Patient Selection and Sample Size Calculation

All control subjects from the previous case-control study [12] were evaluated for inclusion. Eligible study participants were categorised into three groups according to reported khat usage: (i) individuals who never used khat were classified as “nonusers”; (ii) individuals who had stopped chewing khat for more than one year were termed “stopped chewing khat”; and (iii) individuals with current khat use, defined as reported khat usage within the last 12 months, were classified as “khat users.”

### 2.5. Statistical Methods

Categorical variables were summarized as frequencies, whereas continuous variables were presented as median and interquartile range (IQR) since the data were not normally distributed. Comparisons between groups were performed using the Pearson's chi-square test for categorical variables, and Mann–Whitney U test for continuous variables. Khat users were further categorised as “heavy users” or “light users” according to the median lifetime khat exposure measured in* khat-years*. In the multivariable analysis a logistic regression model was used to control for confounders.

The statistical analyses were performed in SPSS 25.0 (SPSS Inc., Chicago, IL, USA). All tests were two-sided and a* p*-value <0.05 was considered significant throughout the study. The* Strengthening the Reporting of Observational studies in Epidemiology* (STROBE) statement guidelines were followed [26].

### 2.6. Ethics

The study was approved by the National Research Ethics Review Committee (NRERC, Ref. No. 3.10/829/07) in Ethiopia and by the Regional Committees for Medical and Health Research Ethics (REK Sør-Øst Ref. No. 2014/1146) in Norway. The study was conducted in accordance with the Declaration of Helsinki [27]. Written informed consent was obtained from all study subjects.

## 3. Results

### 3.1. Study Population

**Figure 2:**
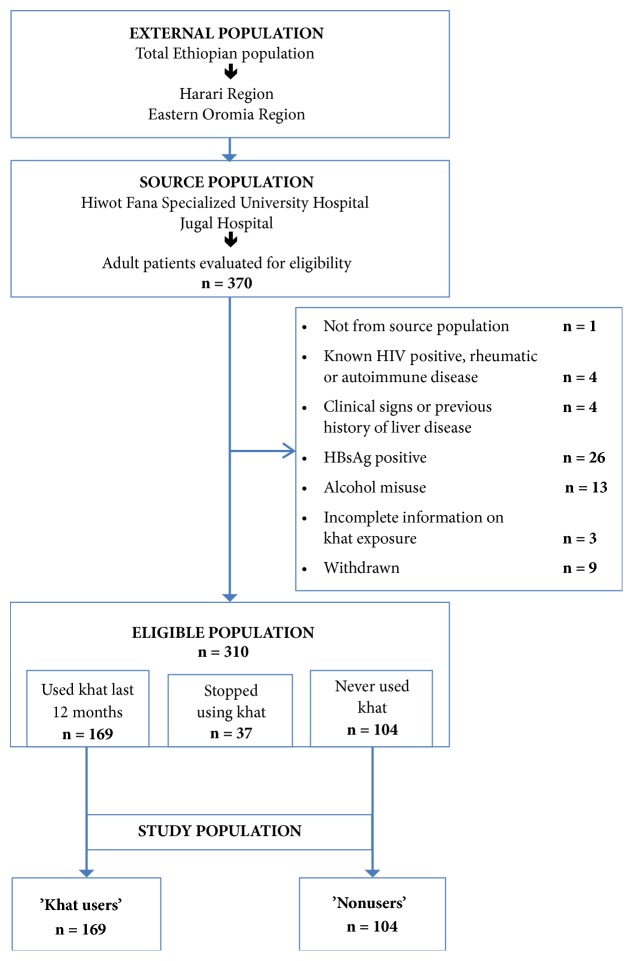
**Study flow diagram illustrating the selection of the study subjects. Abbreviations: HBsAg**: hepatitis B surface antigen;** HIV**: human immunodeficiency virus.

A total of 370 individuals were recruited and evaluated for eligibility, of whom 310 fulfilled the inclusion criteria. Of these, 169 study subjects had a history of using khat within the last 12 months, and 104 had never chewed khat. Thus, the final study population comprised 169 “khat users” and 104 “nonusers” (Figure 2).

### 3.2. Demography

Overall, there were more men (57.5%) than women among the study participants, and the median age was 30 (IQR 24-50) years (Table 1). Study subjects in the nonuser group were younger than the “heavy khat users” (>15* khat-years*) (median 27* vs*. 40 years of age;* p*<0.001). The khat users were more likely than the nonusers to be male, ethnic Oromo, Muslim, and farmers. Overall, men were more likely to have a history of khat use within the last 12 months than women (74.5%* vs.* 44.8%;* p*<0.001). Moreover, among the khat users, men reported higher khat exposure than women (median 23* vs.* 4* khat-years*;* p*<0.001).

### 3.3. Laboratory Findings

Overall, median serum ALT activity was 23 U/L (IQR 17-32), median serum AST activity was 28 U/L (IQR 21-38), and median serum ALP activity was 83 U/L (IQR 66-106) (Table 2). Serum liver transaminase and ALP activities were increased amongst heavy khat users compared to nonusers (ALT: median 25* vs.* 22 U/L,* p=*0.015; AST: median 32* vs.* 26 U/L,* p*=0.003; ALP: median 92* vs.* 77 U/L,* p*=0.003); however, by definition, none of the study participants presented with clinical signs of liver injury or recognized liver disease. When comparing study subjects with circulating autoimmune markers to seronegative study subjects, there was no difference in the proportions with elevated liver transaminase activity (20.2%* vs.* 22.5%;* p*=0.655). Serum ALP activities were increased amongst AMA positive compared to AMA negative (ALP: median 95* vs*. 80 U/L,* p*=0.001); however, none of the study subjects had ALP above the upper reference range [28]. The overall median serum level of IgG was 15.1 g/L (IQR 12.8-17.4) and only four (1.5%) had IgG above the upper reference range [29], with no significant differences between the groups (nonusers: 1.9%* vs. *khat users: 1.2%;* p*=0.984).

The overall proportion of study subjects with circulating autoantibodies was 2.6% for ANA, 15.4% for SMA and 25.6% for AMA. None of the study subjects with a positive ANA had presence of SMA or elevated serum IgG. Khat-users were more likely than nonusers to be ANA positive (4.1%* vs.* 0%;* p*=0.047); however, there was no significant difference in the seropresence of ANA between heavy users and nonusers (3.6%* vs.* 0%;* p*=0.086). No significant differences in SMA or AMA seroprevalence between khat users and nonusers were found (SMA: 16.0%* vs.* 14.4%,* p=*0.730; AMA: 24.9%* vs.* 26.9% (p=0.704).

Since there were no observations of ANA seropositive among the nonusers, the outcome variable “ANA positive” could not be included in the multivariable analysis. In multivariable analysis adjusting for age and sex, no significant association between khat use and SMA or AMA was found (Table 3).

## 4. Discussion

In the present study, the overall seroprevalence of ANA was low, whereas a substantial proportion was SMA and/or AMA positive. No significant association between khat use and circulating SMA or AMA was found. In univariable analysis the association between khat and ANA was borderline significant; however, there was no significant difference in the seropresence of ANA between heavy users and nonusers. Since there were no ANA positive observations in the reference group, it was not possible to further explore the association between khat and ANA in multivariable analysis.

Previous case reports have proposed khat-induced autoimmune response causing acute and chronic liver injury in patients of Somali and Yemeni origin with seropresence of ANA and/or SMA [13–15, 30]. In the present study, khat users were more likely to be ANA positive than nonusers, however, numbers were small and the increased proportion of ANA positive khat users compared to nonusers was of borderline significance, and hence the observed association is at best dubious. Although there may be an association between circulating ANA and khat use, ANA is not specific for autoimmune hepatitis but is also found in patients with other autoimmune diseases, viral infections, a wide range of other liver diseases and even in subgroups of healthy subjects [31, 32]. Similar to ANA, SMA also lacks organ-/ and disease specificity, but is still considered the most specific marker of autoimmune hepatitis [31]; the combined seropositivity for ANA and SMA together with elevated serum IgG increase the specificity and the diagnostic accuracy [16, 33, 34].

Our findings weaken the hypothesis that the pathogenetic mechanism of khat-related liver injury is mediated by autoimmune mechanisms, since (i) there was no association between khat use and circulating SMA; (ii) none of the ANA seropositive study subjects in the present study had concurrent seropresence of SMA or elevated serum IgG; and, (iii) there was no association between elevated liver transaminase activities and the selected circulating autoantibodies. Moreover, these findings correspond to our earlier study of 150 patients with chronic liver disease attending the same hospital from where the study subjects in the present study were recruited, of whom only two (1.3%) patients were attributed to autoimmune liver disease [19]. Although only a limited number of liver biopsies was undertaken in the previous study, the histological findings were supportive of toxic liver injury [19] and mirror those observed in animal models [35] and previous case reports of khat-induced liver injury in patients with a mixed clinical picture of autoimmune hepatitis together with histological evidence compatible with toxic origin [14, 36–38]. However, to distinguish drug-induced liver injury with presence of autoantibodies from autoimmune liver disease is difficult, and was beyond the scope of this study. Future studies should explore this further by following up patients with khat-related liver injury and circulating autoantibodies, and study the seropresence of autoantibodies and manifest liver disease after discontinuation of khat use.

In general, autoimmune diseases more frequently affect women than men [39]. Although men tend to develop autoimmune hepatitis at a younger age than women and have a higher relapse rate, men appear to have reduced susceptibility to the development of autoimmune hepatitis and a better prognosis than women [40]. Studies worldwide have found women significantly more frequently SMA positive than men [41–43]. However, in the present study, there were no sex differences in the presence of autoimmune markers, which correspond with the previous study undertaken in Ethiopia [17].

To the best of our knowledge, the survey undertaken by Tsega et al. [17] in the 1970's is the only study on autoimmune markers in Ethiopia, and only scant data on the seroprevalence of autoantibodies among healthy individuals in sub-Saharan Africa are available. In a study of autoantibodies among 152 elderly individuals (median age 66 years) in rural south-west Cameroon, Njemini et al. [41] found that 9% of healthy elderly were ANA positive. In Sierra Leone, a study of 70 women treated for vesicovaginal fistulas were screened for ANA and as many as 28.5% were found positive [44]. Oyeyinka et al. [45] studied the presence of ANA in 111 plasma samples from healthy Nigerians aged 6 to 95 years of age, of whom 4 (3.6%) were positive. Hence, the observed seropositivity of ANA (2.6%) in the present study was low compared to sub-Saharan and worldwide estimates ranging up to around 30% in healthy controls, although different cut-off titres determining ANA as positive have been used [46–49].

The high proportion of study participants with positive SMA and/or AMA in the present study was intriguing. In Cameroon, SMA was found in 9%, while only 0.7% were AMA positive [41]. The global prevalence of SMA in the general population worldwide is estimated to be around 10-12% [41–43, 50]; hence, the observed proportions of SMA positive (15.4%) in the present study were higher than anticipated. Of note, the strikingly high overall prevalence of AMA (25.6%) in the present study was more than twenty-fold the global estimates [51–55]. However, there are wide differences in quantification methods, analytic thresholds and screening assays available and the observed results in the present study might not be directly comparable to other studies. Nevertheless, the proportion with a positive AMA and/or SMA among the Ethiopian study subjects was much higher than the results obtained from routine clinical samples in Norwegian patients using the same assays (personal communication, Dr. Trine Lauritzen). In this study, circulating autoantibodies were detected solely by ELISA techniques, which for AMA is considered the method of choice. Although indirect immunofluorescence (IIF) is traditionally considered the gold standard, ELISA tests are preferred in clinical laboratories over IIF because they are rapid, automated, standardized and objective, and thus considered more reliable for detection of AMA [56]. In addition, the MIT3-based ELISA-assays have been shown to be more sensitive and specific than both IIF and the conventional anti-M2 ELISA targeting only one of the M2-related autoepitopes [57–59]. However, this study was not designed to determine the regional seroprevalence of autoimmune markers and further adequately powered population-based studies are needed to obtain representative estimates; future prevalence-studies should also consider using different methods in determining autoantibodies and thus confirming the observations and further strengthen the results.

On the other hand, the remarkably high seroprevalence of AMA and SMA observed in this present study raises an important question: could it be that the seroprevalence of autoantibodies, in general, is high in sub-Saharan Africa due to an increased exposure to various infectious diseases and/or other environmental triggers of autoimmunity?

It is known that the upper reference range for eosinophil counts and serum IgG in apparently healthy individuals is higher in sub-Saharan African countries [28, 29] and thus may reflect a higher degree of immune activation compared to Europe and North America. Since the serological tests and assays are largely established in developed countries and epidemiological data on autoimmune markers among healthy individuals in sub-Saharan Africa is scarce, is it possible that the conventional cut-off values are inappropriately low for this African population?

AMA is considered the diagnostic hallmark of primary biliary cholangitis (PBC) [31]. To the best of our knowledge, there are no epidemiological studies on PBC in Ethiopia or sub-Saharan Africa available but the prevalence is assumed to be of the lowest in the world [60, 61], which correspond to the findings in our previous study of patients with chronic liver disease in eastern Ethiopia, of whom none had PBC [19]. Moreover, although serum ALP activities were significantly higher among AMA positive compared to AMA negative, none of the study subjects had ALP above the upper reference range [28], and thus we find it less likely that the observed high seroprevalence of AMA represents an epidemic of latent PBC in this apparently healthy study population.

The paradoxical feature of increased immune response but low incidence of autoimmune diseases among African individuals compared to European individuals has been observed for more than 50 years [62]; however, our understanding of the environmental and genetic factors that might contribute to this resistance against autoimmune diseases in Africa is still in its early days [63, 64].

This study had a number of strengths, most importantly that the sample size was large and the study subjects underwent a rigorous quantification of khat usage and state-of-the art testing for autoimmune markers. Study subjects with manifest liver disease, known autoimmune disease, or recognized trigger factors of autoimmunity were excluded to minimise the influence of underlying disease on the autoantibody profile.

The study also has its limitations. Firstly, the ideal study group would be healthy individuals randomly selected from the source-population. However, in lack of a population roster, the study subjects in the present study were selected among inpatients and outpatients from several hospital departments. Although participants with conditions known to influence on autoimmunity were excluded, there might still have been undiagnosed cases of autoimmune disease among the study subjects. However, the prevalence of autoimmune disease in Ethiopia is expected to be low [17], so the confounding effect is likely to be small. Secondly, underreporting or denial of alcohol consumption or other recreational drugs is common. Alcohol consumption has been identified as a protective factor against autoimmune diseases [65, 66], and thus underreporting would, if anything, underestimate the effect of khat exposure. The use of khat in eastern Ethiopia, however, is legal and socially accepted, and its usage less likely to be underreported in this context. Thirdly, a number of other predictors of autoimmunity were not explored. Of note, cigarette smoking may trigger an autoimmune response [67] and smoking habits were not assessed in the present study, and hence we cannot exclude that cigarette smoking might exert confounding effects not accounted for in our analysis. Finally, cross-sectional studies are not designed for testing a hypothesis but may be useful for raising the question of the presence of an association [68].

## 5. Conclusions

In the present study, there was no association between khat chewing and the seropresence of SMA or AMA. ANA was more common among khat users compared to nonusers, but numbers were small and only borderline significant. Our findings weaken the hypothesis that khat-related liver injury is mediated through autoimmune mechanisms. Of note, the seroprevalence of AMA and SMA were strikingly high in this Ethiopian population compared to global estimates, suggesting that diagnostic algorithms for autoimmune liver diseases developed in Europe and North America might lead to misdiagnosis of patients on the African continent.

## Figures and Tables

**Table 1:** Demographic features of the study participants, by khat consumption level.

Variable	Nonusers (n=104)	Light khat users^1^ (n=86)	Heavy khat users^2^ (n=83)	Significance (*p*)	
				Nonusers *vs.*	Nonusers *vs.*
				Light	Heavy
				khat users	khat users
Men	40 (38.5)	47 (54.7)	70 (84.3)	***0.026***	***<0.001***
Age (years)	27 (22-52)	27 (24-40)	40 (30-55)	0.497	***0.001***
Ethnic group:					
Oromo	48 (46.2)	67 (77.9)	78 (94.0)	***<0.001***	
Amhara	42 (40.4)	15 (17.4)	4 (4.8)		***<0.001***
Other	14 (13.5)	4 (4.7)	1 (1.2)		
Religion:					
Islam	44 (42.3)	69 (80.2)	79 (95.2)	***<0.001***	***<0.001***
Christianity	60 (57.7)	17 (19.8)	4 (4.8)		
Occupation:					
Farmer	9 (8.7)	28 (32.6)	65 (78.3)	***<0.001***	***<0.001***
Housewife	26 (25.0)	10 (11.6)	1 (1.2)		
Student	14 (13.5)	10 (11.6)	0		
Public servant	17 (16.3)	3 (3.5)	3 (3.6)		
Health professional	5 (4.8)	3 (3.5)	2 (2.4)		
Other	33 (31.7)	32 (37.2)	12 (14.5)		
Alcohol use^3^	24 (23.1)	17 (19.8)	6 (7.2)	0.581	***0.003***
Khat-years^4^	0	2 (0.5-10)	60 (30-100)	***<0.001***	***<0.001***

Data are presented as number (%) or as median (interquartile range).

(1) ≤15 khat-years^4^

(2) >15 khat-years^4^

(3) ≤20 grams/day in women and ≤30 grams/day in men.

(4) One khat-year was defined as daily use of 200 grams fresh khat for one year.

**Table 2:** Laboratory findings in the study participants, by khat consumption level.

Variable	Nonusers (n=104)	Light khat users^1^ (n=86)	Heavy khat users^2^ (n=83)	Significance (*p*)	
				Nonusers *vs.*	Nonusers *vs.*
				Light	Heavy
				khat users	khat users
ALT (U/L)	22 (16-31)	23 (18-31)	25 (18-34)	0.382	***0.015***
AST (U/L)	26 (19-35)	26 (21-33)	32 (24-47)	0.893	***0.003***
ALP (U/L)	77 (62-101)	80 (64-102)	92 (76-108)	0.720	***0.003***
IgG (g/L)	14.9 (12.8-17.0)	15.2 (12.8-17.1)	15.3 (12.6-18.2)	0.924	0.347
ANA positive	0	4 (4.7)	3 (3.6)	***0.040***	0.086
SMA positive	15 (14.4)	10 (11.6)	17 (20.5)	0.570	0.274
AMA positive	28 (26.9)	20 (23.3)	22 (26.5)	0.563	0.949

Data are presented as number (%) or as median (interquartile range).

*Laboratory reference range: *ALT (8-40 U/L); AST (14-40 U/L); ALP (60-306 U/L); IgG (0.8-27.8 g/L) [28, 29].

(1) ≤15 khat-years^3^

(2) >15 khat-years^3^

(3) One khat-year was defined as daily use of 200 grams fresh khat for one year.

***Abbreviations.*** **ALT**: alanine aminotransferase; **AST**: aspartate aminotransferase; **ALP**, alkaline phosphatase; **IgG**: immunoglobulin G; **ANA**: antinuclear antibodies; **SMA**: antismooth muscle antibodies; **AMA**: antimitochondrial antibodies.

**Table 3:** Association between khat and frequency of circulating SMA or AMA, by khat level.

Variable	Crude OR	Significance	Adjusted OR^1^	Significance
	(95% CI)	(*p*)	(95% CI)	(*p*)
SMA				
Nonusers^2^	1		1	
Light users^3^	0.78 (0.33-1.84)	0.571	0.89 (0.37-2.12)	0.785
Heavy users^4^	1.53 (0.71-3.28)	0.276	1.80 (0.76-4.26)	0.180
AMA				
Nonusers^2^	1		1	
Light users^3^	0.82 (0.42-1.59)	0.563	0.81 (0.42-1.60)	0.551
Heavy users^4^	0.98 (0.51-1.88)	0.949	1.21 (0.58-2.52)	0.608

(1) Adjusted for the confounding effects of age and sex.

(2) Reference group

(3) ≤15 khat-years^5^

(4) >15 khat-years^5^

(5) One khat-year was defined as daily use of 200 grams fresh khat for one year.

***Abbreviations.* OR**: odds ratio; **CI**: confidence interval; **SMA**: antismooth muscle antibodies; **AMA**: antimitochondrial antibodies.

## Data Availability

The data used to support the findings of this study are available from the corresponding author upon request.

## List of Abbreviations

ALP: Alkaline phosphatase
ALT: Alanine aminotransferase
AMA: Antimitochondrial antibodies
ANA: Antinuclear antibodies
anti-HCV: Hepatitis C virus antibody
AST: Aspartate aminotransferase
CI: Confidence interval
ELISA: Enzyme-linked immunosorbent assay
HBsAg: Hepatitis B surface antigen
IgG: Immunoglobulin G
IQR: Interquartile range
OR: Odds ratio
SMA: Antismooth muscle antibodies.
